# Real Time Apnoea Monitoring of Children Using the Microsoft Kinect Sensor: A Pilot Study

**DOI:** 10.3390/s17020286

**Published:** 2017-02-03

**Authors:** Ali Al-Naji, Kim Gibson, Sang-Heon Lee, Javaan Chahl

**Affiliations:** 1 School of Engineering, University of South Australia, Mawson Lakes, SA 5095, Australia; Sang-Heon.Lee@unisa.edu.au (S.-H.L.); Javaan.Chahl@unisa.edu.au (J.C.); 2 Electrical Engineering Technical College, Middle Technical University, Al Doura 10022, Baghdad, Iraq; 3 School of Nursing and Midwifery, University of South Australia, Adelaide, SA 5001, Australia; Kim.Gibson@unisa.edu.au; 4 Joint and Operations Analysis Division, Defence Science and Technology Group, Melbourne, Victoria 3207, Australia

**Keywords:** apnoea, apparent life-threatening event, Microsoft Kinect sensor, real-time image sequence analysis, motion magnification, motion detection

## Abstract

The objective of this study was to design a non-invasive system for the observation of respiratory rates and detection of apnoea using analysis of real time image sequences captured in any given sleep position and under any light conditions (even in dark environments). A Microsoft Kinect sensor was used to visualize the variations in the thorax and abdomen from the respiratory rhythm. These variations were magnified, analyzed and detected at a distance of 2.5 m from the subject. A modified motion magnification system and frame subtraction technique were used to identify breathing movements by detecting rapid motion areas in the magnified frame sequences. The experimental results on a set of video data from five subjects (3 h for each subject) showed that our monitoring system can accurately measure respiratory rate and therefore detect apnoea in infants and young children. The proposed system is feasible, accurate, safe and low computational complexity, making it an efficient alternative for non-contact home sleep monitoring systems and advancing health care applications.

## 1. Introduction

Cessation of respiration for 20 s or more, or a shorter pause resulting in cyanosis, obvious pallor or hypotonia or a marked decrease in heart rate with no respiratory, defines central apnoea [1]. Central apnoea is characterized by a lack of controlling breathing during sleep due to failure of the brain to correctly signal the muscles responsible for breathing. Other forms of apnoea include obstructive and mixed aetiologies, where inspiratory effort still occurs but without effective air flow and will not be discussed further in this work. Home apnoea monitoring may be an appropriate intervention for some children. Whilst there is no scientific evidence that a home apnoea monitor will prevent sudden unexpected infant death, including sudden infant death syndrome [1,2], there are situations where home monitors may be warranted. These circumstances include premature infants who have a prolonged course of apnoea (prolonged pauses in breathing) and bradycardia after discharge from hospital and are generally monitored until 43 weeks postmenstrual age when this most likely resolves. Infants who have medical conditions affecting breathing regulation or have unstable airways, infants who are technology-dependent, such as those requiring home respiratory support, have tracheostomies or oxygen therapy with chronic lung disease, or infants who have experienced an apparent life-threatening event (ALTE) requiring significant resuscitation may also be discharged home from hospital with an apnoea monitor [1,3].

Current methods for apnoea monitoring can be problematic. The most widely used form of non-invasive home respiratory monitoring for infants is with transthoracic impedance (TTI). This contact method requires the application of standard electrocardiogram (ECG) leads or two electrodes placed on the thorax and secured by a chest belt, and regular infant movement can disrupt the signal. Constant false alarms generated from this technology may cause significant parental frustration and associated non-compliance [4]. Monitors with leads may also increase the risk of strangulation or entrapment of the infant [5]. Home apnoea monitors are also expensive, with an estimated monthly cost of $300–$400 US [6]. Therefore, there is a significant need to provide a reliable remote monitoring system to observe respiratory activity in infants and young children, particularly in home applications. Several researchers have performed a variety of remote methods for respiratory monitoring, including methods based on radar effect [7,8,9,10]. However, using Doppler effect in sleep monitoring requires specialist hardware to produce radar output signals and receive a reliable return signal. Current studies suffer significant signal to noise ratio (SNR) decreases at distances larger than 1 m between the subject and the antennas due to increased free space loss [11]. Furthermore, the radar antenna must be directed towards the chest wall and any irregular movement of the subject leads to produce noise [7,8,9,10]. In addition, focused radar energy may be discovered to have harmful side effects on biological tissue [12]. A study by Yang et al. [13] proposed a portable wireless monitoring system for sleep apnoea detection based on active radio frequency identification (RFID) technology. Although this study was designed to consume low power and reduce a cost of the overall system, it is still needed to place the sensors on the patient’s body which may lead to discomfort when sleeping. In addition, the transmitted signal from on-body sensors to the RFID reader may be distorted by the patient’s movement and signal interference. Another study by Yang et al. [14] presented a novel wireless transducer based on analogue technology for remote monitoring under a single sleeping scenario for one participant without any physical contact. Their study provided a continuous sleeping monitoring, improving patient comfort and minimizing healthcare costs. However, amplitude modulating, filtering, amplifying and separating analogue signals needs to be considered carefully when the noise signal resulting from patient’s movement and signal interference falls within the working frequency. Another study by Jones et al. [15] used pressure sensors placed above and below the bed to monitor respiratory activity without any restrictions for the patient. However, a limitation of this is that measurements are also affected by patient movement. Non-contact methods based on electromagnetic sensors have also been developed for sleep monitoring [16,17,18]. Electromagnetic sensors have the advantage of not requiring adhesive sensors and electrodes and the signal is also un-attenuated by bone and skin. However, these methods require subjects to remain stationary and are limited in their range [17]. Thermal video cameras have been used in several studies [12,19,20] to monitor respiratory activity by detecting carbon dioxide emissions or by determining skin temperature differences through inspiration and expiration. Although thermal cameras were attractive to monitor respiratory rates, its measurements are also affected during head rotation, irregular movements, and particularly any apparatus that covers the face. Thus, thermal cameras are unable to determine respiratory activity when the nasal region is not clear to analyze. Some studies [21,22,23] used an image sequence analysis captured by video camera as a remote methods to measure respiratory activity by analyzing optical flow of chest surface movements resulting from respiration. Because these studies relied on optical flow calculations, computational complexity, motion artefacts, lighting conditions were the main drawbacks. Also, an image sequence analysis based on motion magnification system was recently used by Al-Naji and Chahl [24] to measure respiratory activity for a baby in different sleep positions. Although this study succeeded to detect the respiratory rate and breathing time parameters in different positions (even in the presence of the blanket), the data had to be interpreted after video recordings were analyzed and abnormal events could not be captured in real time. Other studies [25,26] used 3D surface information of the patient’s chest and abdomen captured by time of flight (ToF) cameras for detecting the respiratory motion. The studies are based on time variation of the signals obtained by averaging the range values in two fixed points. Though using 3D surface information is conceivable in theory, the measurements are determined by a distance between the patient and the camera and they are somewhat affected by noise and motion artefacts and ToF cameras are expensive. Based on photoplethysmography imaging (PPGI) signals, several researchers [27,28,29,30] have used camera-based PPGI signals to determine variations in the skin blood volume resulting from cardiorespiratory rhythms. Previous studies were affected by lighting conditions, skin tone, and distance [28] which may cause background noise falling within the frequency band of interest. These methods cannot be used to detect respiratory activity in unclear regions of interest (ROIs); therefore, PPGI cannot operate on a subject that changes to different positions.

To overcome the problems under these assumptions, this paper aims to propose a new real-time vision system based on Kinect sensor (developed by Microsoft based in Redmond, WA, USA) to monitor respiratory activity in any given sleep position, light conditions (even if installed in a dark environment) and whether the subject is covered or not, while being reliable, safe and cost effective. Furthermore, the proposed system has utilized several improvements, including wavelet decomposition, image de-noising and resampling to enhance performance beyond standard video magnification systems in terms of noise removal, video quality and execution time which make it suitable for real-time use.

The rest of the paper is organized as follows: Section 2 reviews some of the previous relative works based on Kinect sensor. Section 3 presented a description of the Kinect sensor. In the next section, materials and methods are provided. The results and discussion are presented in Section 5 and finally, Section 6 concludes the paper.

## 2. Related Works

Recently, there has been interesting research using a Kinect sensor as a non-contact device for tracking and detecting respiratory activity. For example, Xia and Siochi [31] proposed a respiratory monitoring system based on Kinect v1 sensor. This study used depth images captured by the Kinect sensor to determine the average depth over a thoracic region of interest. The ROI was manually determined by placing a translation surface on the patient’s thorax in the center of the image. Although this study was implemented in real-time, it required a clear ROI for measurement. Other studies [32,33] presented a sleep monitoring system using a Kinect v1 sensor, where thorax movements are detected by tracking over time the depth information recorded during sleep. Because there is no ROI tracking system in these studies, ROI must be in the center of the image and any unexpected movement leads to distorting and biasing the results. Also, some studies used a Kinect sensor to analyze the breathing activity and sleep disorders based on depth map information recorded during patient sleep while facing the Kinect sensor [34,35,36,37]. However, some limitations were related to unclear ROI and subject movement during the measurement. Another study by Harte et al. [38] proposed a developed system for analyzing chest wall motion based on four Kinect sensors. The sensors were placed around the subject with a distance of 1 m to create a 3D time-variation view of the patient’s torso. A benefit of using data from four Kinect sensors is to analyze chest wall motion even with moving subjects which may be useful in scenarios such as when measuring dynamic hyperinflation during exercise. However, this study did not focus on the movement of the diaphragm as well as being prone to some errors in the 3D reconstruction due to a design limitation of the sensors that are unsynchronized in time and frequency. Other studies [39,40] utilized a Kinect-based system to solve issues related to patient set-up misalignment and respiratory motion during radiotherapy. They used depth map information for patient set-up and breathing motion management using several ROIs within the abdominal-thoracic area. However, there is only one accurate position, which is for a subject to face the Kinect sensor and any movement would lead to further sources of error. A study by Lee et al. [41] proposed a sleep monitoring system based on a Kinect v2 sensor to detect the sleep patterns and postures during sleep without any attached devices. They used depth information to detect whole human body joints to gather the sleep movement, posture, and sleep information. However, it is it difficult to differentiate the subject’s front from their back as well as the human body not being able to be covered by a blanket in their study. Our current study is to develop a real-time vision system based on a Microsoft Kinect v2 sensor to detect breathing activity by tracking the interest region located within five joints that corresponds to both chest and abdomen areas. Our study can measure respiratory rates and detect apnoea in any sleep position, regardless of light conditions and whether the subject is covered.

## 3. Kinect Sensor

Kinect v1 was released as a peripheral device by Microsoft (Redmond, WA, USA) in 2010 for gaming XBOX360 purposes and due to the massive demand of the market, Microsoft has developed it to be compatible with Windows via Microsoft’s Kinect Standard Development Kit (SDK) and power adaptor [42,43,44,45]. The next generation of Kinect sensor (Kinect v2) released in 2014 has advantages over the original Kinect technology in terms of performance, accuracy and wide field of view [42,43,44,45]. This is because the Kinect v2 uses a ToF technology [46] for the depth measurements instead of the structured light coding technology [47] used in Kinect v1. A comprehensive comparison between Kinect technologies can be found in [45]. Table 1 summarizes some comparative specifications of Microsoft Kinect v1 and v2.

**Figure 1:**
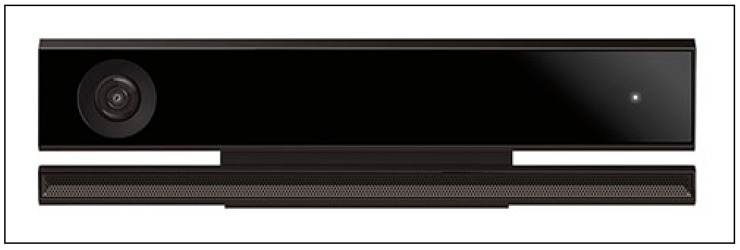
Microsoft Kinect v2 sensor.

Microsoft Kinect v2 has three optical sensors: a RGB camera, IR sensor, IR projector which can provide three outputs; a RGB image, IR image and depth image. It can provide a body tracking, 3D body reconstruction, skeletal tracking, joint tracking and human recognition based on information obtained from the depth and colour sensors at any ambient temperature. Because Kinect v2 is commercially available, has specific features at a low cost, and designed for the sustained commercial use, it is an attractive device for many biomedical applications in both domestic and clinical environments [31,32,33,34,35,36,37,38,39,40,41]. Figure 1 shows the external view of the Microsoft Kinect v2 sensor.

## 4. Materials and Methods

### 4.1. Experimental Setup

Five subjects (three males and two females) with ages ranging from 1 to 5 years, weights from 10 to 17 kg and heights from 75 to 107 cm participated in our sleep monitoring experiments. The required ethical approval was granted by the UniSA Human Research Ethics Committee and it was carried out following the rules of the Declaration of Helsinki of 1975. Written informed consents were obtained from the parents before commencing the experiments. We performed the sleep monitoring in the home environment setup for approximately 3 h for each subject and we repeated the experimentation at different times (during the night and daytime), light conditions (more-lit and dark environment) and subjects with and without a blanket to obtain sufficient data. The Kinect sensor was located to the front of the subject in the direction of 45° at a distance of 2.5 m. The reference methods for monitoring respiration were recorded by a Piezo respiratory belt transducer (MLT1132, ADInstruments, NSW, Australia) and commercial product (Misfit Sleep Monitor, Misfit, Burlingame, CA, USA) for validation purpose. The proposed system is implemented under the MATLAB environment- R2016a (MathWorks, NSW, Australia) with Microsoft Windows 10 operating system, Visual Studio 2013, and Kinect SDK.

### 4.2. System Design/Overview

**Figure 2:**
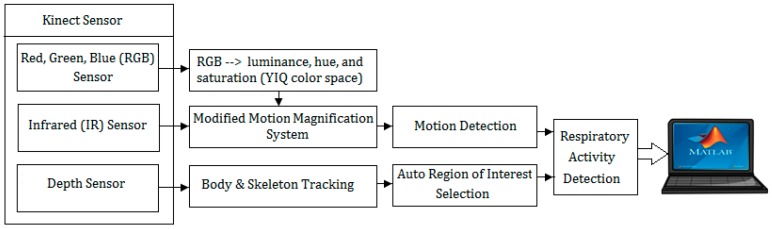
System overview. The proposed system includes a Kinect sensor connected to a laptop via Microsoft Kinect adapter; a real-time system based on motion magnification and motion detection; and software built-in Kinect library, including body index and skeleton tracking.

The overview of the proposed system is presented in Figure 2.

### 4.3. Data Analysis

**Figure 3:**
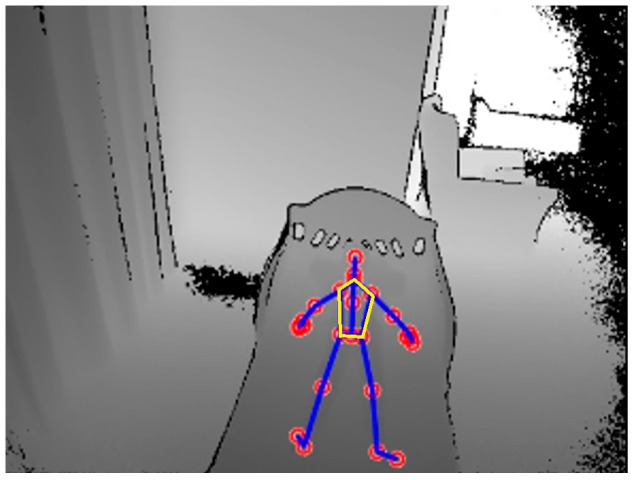
Skeletal joints provided in the Kinect code library. The Region of interest is the yellow pentagon defined by the 5 points.

A Microsoft Kinect v2 sensor was used in this study to detect and track the movement of chest and abdomen caused by inhalation and exhalation. Based on depth information, the Kinect v2 sensor tracks movements of the human body by determining the position of 25 skeletal joints as shown in Figure 3.

We tracked the area within five joints that corresponds to both chest and abdominal areas. This area is determined by tracking five joints, which includes left and right shoulder joints, spine shoulder joint and left and right hip joints. We stored the *x*, *y*, and *z* positions of these five joints by modifying the respective joint functions provided in the Kinect library and used them to determine an auto ROI.

#### 4.3.1. Modified Motion Magnification System

To provide a real-time magnification, better noise performance and to support magnification factors better than linear Eulerian video magnification (EVM), a wavelet pyramid decomposition [48,49] and image de-noising [50] based on linear averaging filter were used to enhance performance of standard video magnification techniques [51,52]. The details of main equations and how the video magnification technique works can be found in [51,52,53].

In the modified motion magnification system, frame sequences of the RGB camera are converted to YIQ channel to separate the intensity information from the colour information. Only Y channel is resized down by using the Lanczos resampling method [54] to reduce processing time. The Y channel is then decomposed into different spatial frequency bands using wavelet pyramids. A temporal band-pass filtering is applied on each level of the wavelet pyramid to extract frequency bands of interest. The extracted band-passed signals are then multiplied by a magnification factor (M) to amplify signals of interest, and these signals are collapsed to obtain the magnified signals. The magnified signals are then filtered based on image de-noising [50] to increase the signal to noise ratio and resize them back to the original size. The magnified signals are then added back to the input signals to obtain processed Y channel. The processed Y is then concatenated with the original I and Q channels along array dimension and converted to RGB to obtain the final output. Since RGB sensor is not affective in the dark environment, the modified motion magnification system is also applied on the frame sequences obtained from IR Kinect sensor in the less-lit environment.

#### 4.3.2. Motion Detection Based on Frame Differencing

The objective of motion detection based on frame differencing is to extract a respiratory motion in the selected ROI in consecutive video frames to recognize the presence of breathing. Basically, frame differencing method suggests that a pixel is moving if its intensity has significantly changed between two consecutive frames as follows [55]:

(1) $$\left|I_{t}\left(i,j\right)-I_{t-1}\left(i,j\right)\right|\geq \tau$$

where $I_{t}\left(i,j\right)$ and $I_{t-1}\left(i,j\right)\;$ represent image intensities for current and previous frames respectively and τ corresponds to a threshold that describing a significant intensity change. If the difference is greater or equal than τ then it is considered as a pixel caused by breath movement, otherwise it is considered as a no-breath movement pixel. The threshold range is from 0 to 255.

### 4.4. Respiratory Calculations

After determining motions above threshold τ in the ROI in all frame sequences caused by respiratory movement, a binary thresholding operation followed by a sequence of binary morphological filters was performed to separate the pixels corresponding to the breath movement to that without movement. This resulted in a binary image, thresholds at ≥110 were set to generate binary images, where the pixels corresponding to changes from breath movement were set to one and the pixels corresponding to areas unchanged by breath movement were set zero. The binary matrix was converted into a binary vector with values 0 and 1, where 0 represents the dark area and 1 represents the white area in the image. Since the Kinect sensor operates with a frame rate of 30 fps, we can find the frame interval using:

(2) rame interval = 1/fps = 1/30 = 0.0333 s

Respiratory rate readings are not instantaneous because they rely on calculating a difference between two consecutive breaths in the frame sequences (N). Let $A_{i}$ be a vector of length N for a number of consecutive breaths:

(3) $$A_{i}=\;\left[A_{i}\left(1\right),\;A_{i}\left(2\right),\;A_{i}\left(3\right),\;\ldots \ldots ..\;A_{i}\left(N\right)\right]$$

where $A_{i}$ is a binary vector that contains values 0 and 1, where zeroes represent the frame sequences without detected respiratory motion and ones represent the frame sequences with detected respiratory motion.

To determine differences between adjacent elements of $A_{i}$, let $B=diff\left([0\;A_{i}\right])$ return a vector of length N−1. To determine positions of a nonzero value in B, we apply C=find(B) that returns a vector with M length containing nonzero values. By calculating the differences among C values, and multiply them by the frame interval, we can measure the vector of respiratory cycles $\left(R_{c}\right)\;$ over time (t). This vector is stored in the Matlab workspace. For the next N sequence, we repeat the previous steps to obtain other respiratory cycles and so on. Now we can detect apnoea and respiratory rate (breaths/min) using the following relations:

(4) $$Rc_{current}\;=Rc_{2}-Rc_{1}=\{\begin{array}{c} Rc_{current}\approx \;Rc_{1},\;Rc_{2},\;\ldots Rc_{M-1}\;\Rightarrow normal\\ \;Rc_{current}\;\geq \;10sec⟹Apnea \end{array}$$

(5) $$respiratory\;rate\;=\;\frac{60\;s}{Rc}$$

Generally, the respiratory rate varies with age, but the normal range is between 30–60 breaths/min for infants, 22–28 breaths/min for children, 16–20 breaths/min for teenagers and 14–18 breaths/min for adults [56]. A pre-set alarm function is considered in this study when the results fall outside theses ranges.

## 5. Results

**Figure 4:**
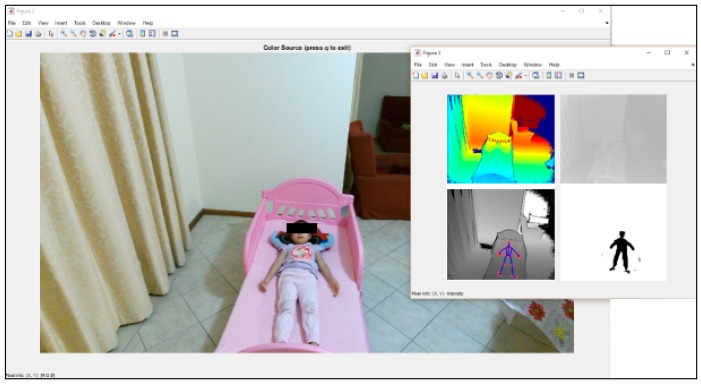
Red, green & blue image, depth map, thermal, skeleton tracking, and body index from the Kinect v2 sensor.

The capturing of RGB, depth, IR, skeleton tracking and body index from the Kinect v2 sensor is shown in Figure 4.

**Figure 5:**
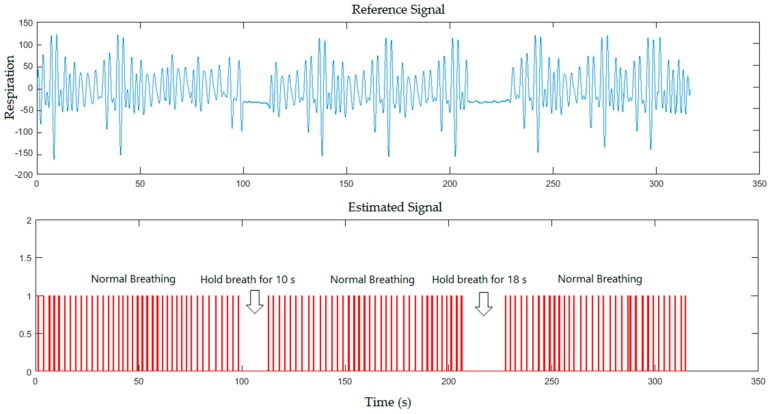
A five minutes simulation for healthy subject where the subject was asked to hold his breath twice during the measurement for the first scenario (without a blanket).

The experimental results obtained from five subjects at many positions were set in two scenarios. The first scenario is when the experiments were carried out in a more-lit environment. The frames sequence in this state obtained from RGB sensor were processed through the proposed system. The second scenario is a less-lit environment (or dark environment). The frames sequences in this state obtained from IR sensor were processed. The experimental results for both scenarios were implemented with and without a blanket. Because all participants in this study are apparently healthy, we asked the participants to hold their breaths during the measurement to create a situation mimicking Apnoea. In the first scenario, the real-time breathing simulation signal based on frame sampling time of five minutes was compared to the reference signal for healthy subject, where the subject was asked to hold his breath for 10 s and 18 s during the measurement in a normal illumination level as shown in Figure 5.

**Figure 6:**
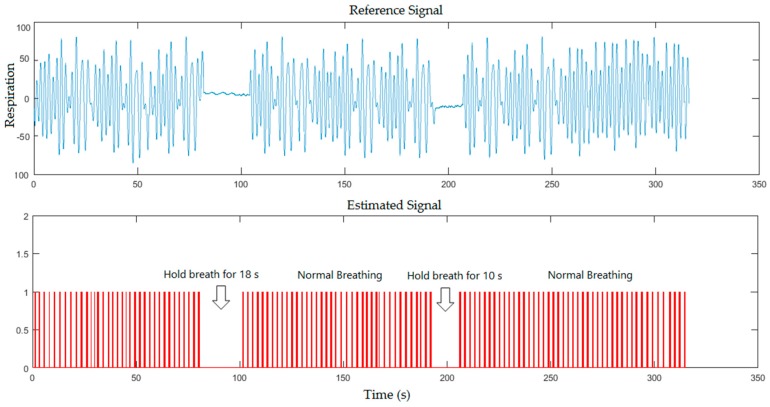
A five minutes simulation for healthy subject where the subject was asked to hold his breath twice during the measurement for the first scenario (with a blanket).

The breathing simulation signal against the reference signal is also shown in Figure 6 when the subject was covered by a blanket and asked to hold his breath for 18 s and 10 s during the measurement in the same illumination level.

**Figure 7:**
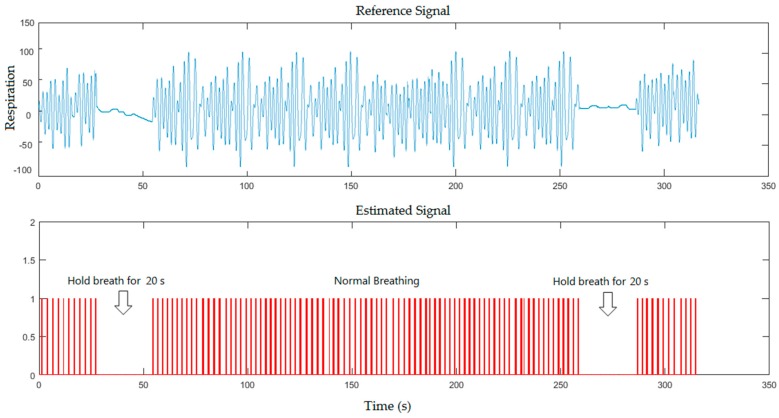
A five minutes simulation for healthy subject where the subject was asked to hold his breath twice during the measurement for the second scenario (without a blanket).

**Figure 8:**
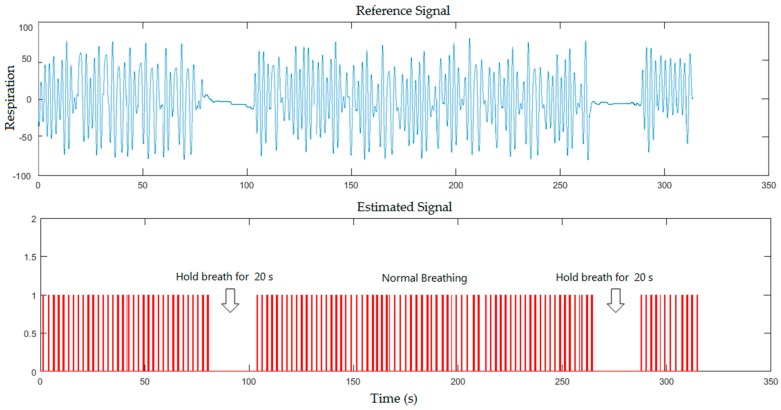
A five minutes simulation for healthy subject where the subject was asked to hold his breath twice during the measurement for the second scenario (with a blanket).

In the second scenario, the breath-holds signals were compared to the reference signals for healthy subject (with and without a blanket), where the subject was asked to hold his breath twice for 20 s during the measurement as shown in Figure 7 and Figure 8 respectively.

From the previous figures, the proposed system could detect the periods of simulated apnoea for 10 s that approximately corresponds to 300 frames (10 s/0.0334), 18 s that approximately corresponds to 539 frames (18 s/0.0334) and 20 s that approximately corresponds to 599 frames (20 s/0.0334). Our system recognised periods of simulated apnoea and measured respiratory rates using Equations (3) and (4) respectively. Breaths per minute can be determined by multiplying a corresponding number of frames with sampling period between the video frames shown in Equation (2), which located within 1796 frames (60 s/0.0334). We calculated number of pulses in 60 s to determine the respiratory rate per minute. The respiratory rate measurements from Figure 5, Figure 6, Figure 7 and Figure 8 were 24, 26, 25, 26 breaths/min, respectively, against 23, 24, 24, 24 breaths/min obtained from the reference data.

**Figure 9:**
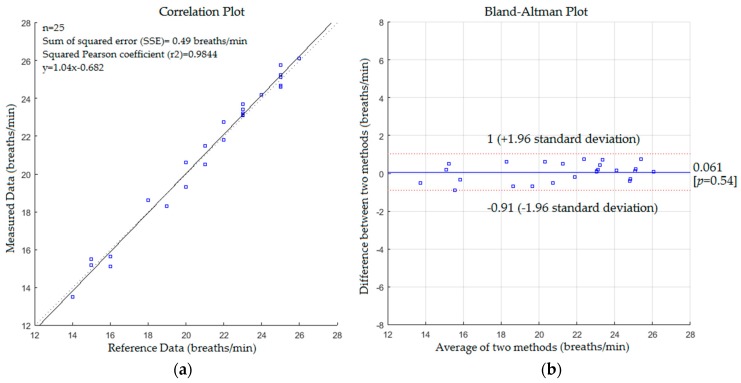
(**a**) Correlation plot; (**b**) Bland-Altman plot of the difference between measured data and reference data (first scenario without a blanket).

The statistical analysis based on correlation plot and Bland-Altman method [57] was used to measure the variability of the proposed system with the reference method. The respiratory data was assessed by recording the spontaneous respiratory signal for each subject using a Piezo respiratory belt attached to PowerLab-based computer (ADInstruments, NSW, Australia) which performs analogue to digital conversion and recording of the time-series signal. The respiratory rate values obtained by the Piezo respiratory belt were extracted by calculating a number of peaks in the time-series signal and used as references to compare with those obtained by the proposed system at the same time. The agreement was evaluated using the mean difference (bias) between the measurements, and the 95% limits of agreement (bias ± 1.96 SD) where SD is the standard deviation of the mean of the differences in paired measurements. The statistical analysis based on correlation plot and Bland-Altman method for respiratory rate measurements in the first scenario without a blanket is shown in Figure 9.

It is clearly observed from Figure 9 that there is a positive linear relationship between the measured values and reference data with a slope, intercept and sum of square error (SSE) of 1.04, −0.682 and 0.49 respectively. The correlation coefficients (Pearson correlation coefficient and Spearman’s Rho coefficient) were 0.9922 and 0.9859 respectively.

**Figure 10:**
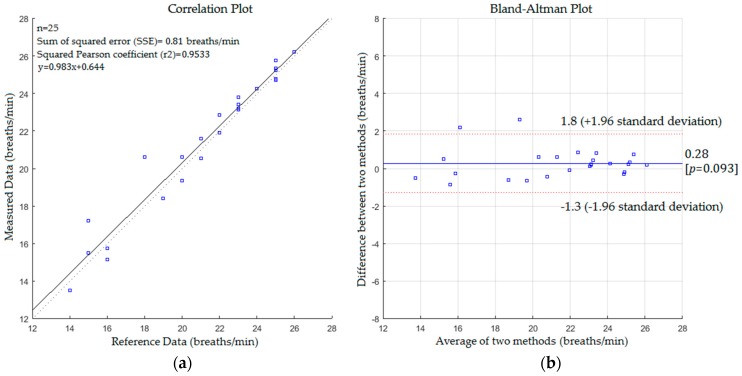
(**a**) Correlation plot; (**b**) Bland-Altman plot of the difference between measured data and reference data (first scenario with a blanket).

**Figure 11:**
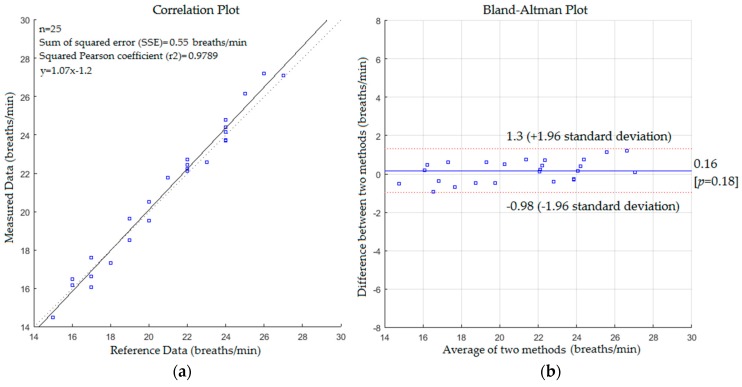
(**a**) Correlation plot; (**b**) Bland-Altman plot of the difference between measured data and reference data (second scenario without a blanket).

The mean difference of the Bland-Altman plot was 0.061% and 95% limits of agreement were −0.91 and +1 respectively with a reproducibility coefficient of 0.97 breaths/min, whereas the statistical analysis for respiratory rate measurements with a blanket is shown in Figure 10. Figure 10 presents a positive linear relationship between the measured values and reference data. The slope, intercept and SSE were 0.98, 0.64 and 0.81 respectively with 0.9764 and 0.9781 of Pearson and Spearman coefficients respectively. The mean difference was 0.28 and 95% limits of agreement were −1.3 and +1.8 with a reproducibility coefficient of 1.6 breaths/min. The results of the statistical analysis for the second scenario without a blanket are presented in Figure 11.

**Figure 12:**
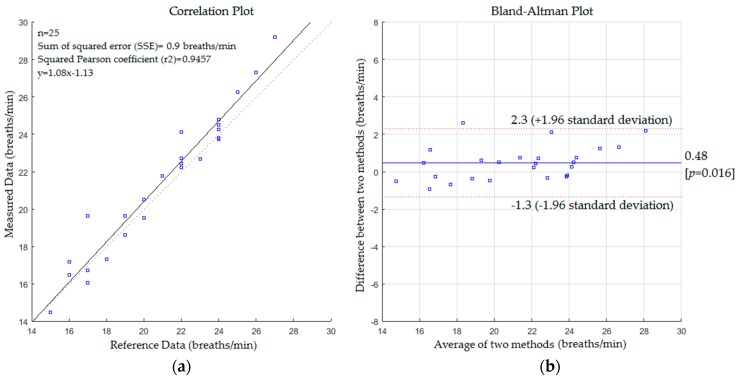
(**a**) Correlation plot; (**b**) Bland-Altman plot of the difference between measured data and reference data (second scenario with a blanket).

As shown in Figure 11, the slope, intercept and SSE were 1.07, −1.2 and 0.55 respectively with 0.9894 and 0.9832 of Pearson and Spearman coefficients respectively. The mean difference was 0.16% and 95% limits of agreement were −0.98 and +1.3 with a reproducibility coefficient of 1.1 breaths/min, whereas the statistical analysis for respiratory rate measurements with a blanket is shown in Figure 12.

Figure 12 presents a positive linear relationship between the measured values and reference data. The slope, intercept and SSE were 1.08, −1.13 and 0.9 respectively with 0.9725 and 0.9634 of Pearson and Spearman coefficients respectively. The mean difference was 0.48% and 95% limits of agreement were −1.3 and +2.3 with a reproducibility coefficient of 1.8 breaths/min.

Comparing the statistics from Figure 9, Figure 10, Figure 11 and Figure 12 reveals that the proposed system with the first scenario works slightly better than with the second scenario. Also, we noted that the statistics obtained from the subject without a blanket were better than those obtained with a blanket. This is because the proposed system may fail to track the body when it is a fully covered by a blanket (including the head) since the Kinect sensor cannot track the human joints in this situation. However, the resulting cross correlation coefficient of the proposed system for all scenarios was 0.9812 which is considered suitable for biomedical applications. When compared to other studies conducted on respiratory monitoring, our correlation coefficient was better than 0.96 [31], 0.98 [36], 0.8656 [38] and 0.90, 0.93 [39].

## 6. Conclusions

Current apnoea monitors used in the home environment are known to be very frustrating to families due to the regular false alarms that they generate. They are also very expensive. Therefore, we have proposed a real-time monitoring system to calculate respiratory rate and detect apnoea based on a Microsoft Kinect v2 sensor and may be utilized in both home and clinical environments. Our system relies on image information captured by the three sensors built-into the Kinect and analyze them through real-time motion magnification and motion detection to detect respiratory activity. We developed an enhanced video magnification technique to suit real-time applications. The experimental results for five subjects with different ages, sleep poses, and light conditions indicate that our proposed system has the potential to measure respiratory rates and detect apnoea even in dark environments. The experiments for both scenarios (more-lit and dark environment) with and without a blanket showed that the correction coefficient between the measured and reference data was very good (0.9812) and acceptable for biomedical applications. Our system may also be used in the future to detect other vital signs and sleep-disorder anomalies amongst other populations and provide a comfortable sleep environment for all whilst being monitored. We therefore believe that this system could potentially be at the forefront of modern respiratory monitoring technology. Further studies with larger numbers of subjects are clearly needed to confirm these findings. Also, future studies should have more sleep apnoea events to determine the clinical usefulness of the proposed system.

## Figures and Tables

**Table 1:** Comparative specifications of Microsoft Kinect v1 and v2.

Features	Kinect v1	Kinect v2
Depth sensor type	Structured light	Time of Flight (ToF)
Red, Green & Blue (RGB) camera resolution	640 × 480, 30 fps	1920 × 1080, 30 fps
Infrared (IR) camera resolution	320 × 240, 30 fps	512 × 424, 30 fps
Field of view of RGB image	62° × 48.6°	84.1° × 53.8°
Field of view of depth image	57° × 43°	70° × 60°
Operative measuring range	0.8 m–4 m (Default); 0.4 m–3.5 m (Near)	0.5 m–4.5 m
Skeleton joints defined	20 joints	25 joints
Maximum skeletal tracking	2	6
